# Case Report: Co-infection with cat-scratch disease and infectious mononucleosis: a rare clinical entity

**DOI:** 10.3389/fped.2025.1660853

**Published:** 2025-11-25

**Authors:** Wenting Huang, Jingping Yuan, Huihua He

**Affiliations:** Department of Pathology, Renmin Hospital of Wuhan University, Wuhan, Hubei, China

**Keywords:** cat-scratch disease, infectious mononucleosis, *Bartonella henselae*, Epstein–Barr virus, histopathology

## Abstract

**Background:**

Cat-scratch disease (CSD) and infectious mononucleosis (IM) are prevalent infections in children and adolescents. However, the concurrent occurence of these two diseases remains a rare clinical entity. We reported a 13-year-old male who presented with unilateral inguinal lymphadenopathy and fever, and had a history of cat scratch. Based on histopathological findings, special stains, and immunohistochemical analysis, the patient was diagnosed with both CSD and IM.

**Case presentation:**

A 13-year-old male presented with a palpable mass on the left thigh and fever. Radiological findings suggested a diagnosis of reactive lymphadenopathy. Laboratory tests revealed elevated C-reactive protein (CRP) levels and the presence of Gram-negative bacilli. Given these findings, the patient underwent an excisional biopsy of the lymph node. Histopathological examination of the excised lymph node revealed features of reactive lymphadenopathy, characterized by mild follicular hyperplasia and an increased number of mononuclear cells in the periphery. Notably, the lymph node displayed multiple small granular microabscesses located centrally and surrounded by a mantle of histiocytes and neutrophils. Special staining with Warthin-Starry silver stain revealed numerous bacilli within the abscesses, findings consistent with *Bartonella henselae* infection. Furthermore, immunohistochemical analysis showed CD20-positive B cells in the follicular areas and CD30-positive plasmablasts and plasma cells. Epstein–Barr virus (EBV) *in situ* hybridization for Epstein–Barr encoded RNA (EBER) was positive in the B-cell lineage, confirming EBV infection. The lymph node showed a polyclonal B-cell proliferation, encompassing a mature B-cell lineage with plasmablasts and plasma cells, indicating a reactive process rather than malignancy.

**Conclusion:**

This case represents the first reported instance of a pediatric patient diagnosed with concurrent CSD and IM by pathological examination. The diagnosis was based on histopathological features, special stains, and immunohistochemical findings. The coexistence of these two distinct infections highlights the importance of considering multiple etiologies when evaluating reactive lymphadenopathy.

## 1 Introduction

Cat-scratch disease (CSD) is a benign condition caused by *Bartonella henselae* infection, typically manifesting as self-limiting regional lymphadenitis. The bacteria primarily reside in the oral secretions of kittens and can be transmitted to humans through scratches or bites from infected felines. CSD can occur in all age groups, with children and adolescents being the most commonly affected populations. Individuals with compromised immune systems may be more susceptible to infection and prone to developing severe clinical manifestations. Immunocompromised children are hospitalized for cat-scratch disease five times more often than their immunocompetent counterparts and are markedly more susceptible to disseminated infection (1). In immunocompetent children, CSD typically manifests as regional lymphadenopathy. However, approximately 5%–10% of cases may develop atypical presentations such as fever of unknown origin (FUO), hepatosplenic granulomas, or disseminated infection (2). Notably, *Bartonella henselae* represents the third most common infectious pathogen causing FUO in children, accounting for 10.4% of cases (3). Infectious mononucleosis (IM) is an acute viral infection caused by Epstein–Barr virus (EBV), primarily transmitted through saliva and respiratory droplets. IM is most prevalent in individuals aged 10–30 years. The disease is transmitted through direct contact with infected oral secretions, respiratory droplets from coughing or sneezing, or indirect contact with contaminated surfaces.

Studies have suggested that acute viral infections (e.g., EBV) may facilitate *Bartonella henselae* dissemination through transient immunosuppression (4). However, such co-infections remain exceptionally rare in pediatric populations. This paper reported a case of *Bartonella henselae* co-infection occurring alongside an active EBV infection in a 13-year-old male. To our knowledge, this is the first confirmed diagnosis of dual infection established through combined analysis of pathological histomorphology under microscopy and special staining techniques, providing valuable insights into the clinical characteristics, diagnostic challenges, and pathological mechanisms associated with co-infection.

## 2 Case presentation

### 2.1 General information

The patient was a 13-year-old adolescent male who presented with a left medial thigh mass incidentally discovered during bathing 6 days earlier, accompanied by persistent fever with a maximum temperature of 39.1 °C. Physical examination revealed no tonsillar enlargement, and pulmonary auscultation revealed no crackles or wheezes. The patient had previously been healthy, with no history of chronic or infectious disease. On examination, the mass demonstrated a moderately firm consistency with mild erythema and swelling, showing no ulceration. The patient reported mild spontaneous pain that intensified upon palpation. Ultrasonography and magnetic resonance imaging (MRI) revealed a well-defined mass, suggestive of an enlarged lymph node.

The patient was admitted to the Department of Internal Medicine at our hospital for etiological investigation due to FUO accompanied by a leg mass. Laboratory tests revealed negative IgM antibodies for common respiratory pathogens. Fungal infection was excluded through serum (1,3)-β-D-glucan test (result: 52.071 pg/mL, reference range: 0–70 pg/mL) and fluorescent staining smears. Additionally, the serum galactomannan test for *Aspergillus* infection (result: 0.097, reference range: 0–0.49) was negative, and preliminary tumor-marker screening yielded negative results. However, laboratory tests revealed elevated C-reactive protein (CRP) levels (result: 15.39 mg/L, reference range: 0–10 mg/L). And a procalcitonin quantitative assay indicated the presence of a bacterial infection (result: 0.17 ng/mL, reference range: 0–0.05 ng/mL), and Gram-positive cocci and gram-negative bacilli were identified in the tissue fluid smear. For definitive diagnosis and treatment, the patient was transferred to the Department of Surgery for mass excision.

### 2.2 Histopathological findings

**Figure 1:**
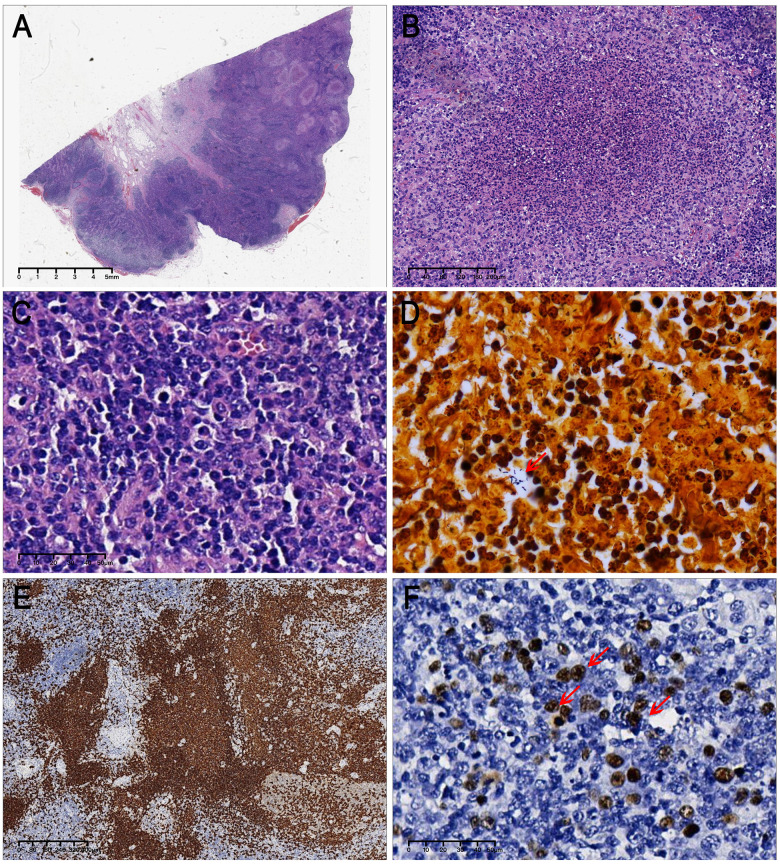
Histopathological features of the case. **(A)** The lymph node demonstrated an intact capsule, preserved lymphoid sinus, and normal follicular architecture, with numerous central eosinophilic microabscess and follicle-surrounding monocytoid cell hyperplasia. **(B)** The microabscess demonstrated central accumulation of proliferative neutrophil debris and fibrinoid material, circumscribed by histiocytes to form pyogenic granuloma. **(C)** Prominent lymphoid hyperplasia with pleomorphic lymphocytes was noted in the extra-cortical germinal centers, exhibiting a lineage distribution of immunoblasts, plasmablasts, and plasma cells within the nodal parenchyma. **(D)** Warthin-Starry silver staining revealed numerous bacilli. **(E)** CD20 immunostaining highlightsed parafollicular hyperplasia of monocytoid B cells. **(F)** EBER-positive cells of varying sizes exhibited a distribution pattern consistent with B-cell maturation lineage morphology.

Postoperative pathological examination identified the mass as lymph node tissue. Under low-power microscopy, the lymph node capsule appeared intact with preserved lymphoid sinus architecture and normal follicular structures with only mild follicular hyperplasia (Figure 1A). Higher-power views revealed numerous centrally eosinophilic microabscesses within the lymph node parenchyma, along with increased monocytoid-cell proliferation around the follicles. Under high-power magnification, two characteristic features were identified. First, microabscess structures were observed, consisting of aggregated neutrophil debris and fibrinoid material within eosinophilic centers, which were surrounded by histiocytes forming pyogenic granulomas (Figure 1B). Second, extensive lymphoid hyperplasia of variably sized lymphocytes was observed outside the cortical zone germinal centers (Figure 1C). Morphologically, large cells with prominent nucleoli consistent with immunoblasts were present, along with smaller cells containing eccentric nuclei typical of mature plasma cells. Intermediate-sized cells displaying transitional morphology between these populations were identified as plasmablasts. Thus, these proliferating cells exhibited polyclonal hyperplasia with a full maturation spectrum, suggesting a reactive condition.

### 2.3 Immunohistochemical analysis and special staining

For pathogen identification in pyogenic granuloma, meticulous examination using Warthin-Starry silver staining under high magnification revealed numerous bacilli within the pyogenic granuloma lesions (Figure 1D). Additionally, the patient's procalcitonin level was 0.17 ng/mL (reference range: 0–0.05 ng/mL). Considering the history of cat scratch exposure prior to symptom onset, a comprehensive diagnosis of CSD was established.

Histopathological morphology combined with special staining, clinical examinations, and medical history supported a diagnosis of pyogenic granuloma secondary to CSD. However, parafollicular monocytoid cell hyperplasia was identified in these lesions—an atypical feature in classic CSD, warranting further histological analysis. Immunohistochemistry demonstrated CD20 positivity in B lymphocytes within germinal centers and the surrounding mantle zone. Notably, CD20 staining confirmed that proliferating monocytoid cells adjacent to follicles in H&E-stained sections were monocytoid B cells (Figure 1E). CD3 staining showed normal positive expression of T lymphocytes in the interfollicular areas, with appropriately scattered follicular helper T cells within germinal centers. Ki-67 showed a normal proliferation index. CD30 staining revealed scattered positive immunoblasts. *in situ* hybridization demonstrated EBV-encoded RNA (EBER) in cells of variable size (Figure 1F), with approximately 3% of cells positive. Under high-power magnification, these positive cells exhibited morphologies consistent with immunoblasts and monocytoid B cells, aligning with the maturation and differentiation trajectory of B lymphocytes. This pattern indicates heterogeneous proliferation of EBV-infected immunoblasts, plasmablasts, and plasma cells. Based on the histomorphological features, immunohistochemical findings, *in situ* hybridization results, and the clinical manifestations of fever and lymphadenopathy, the diagnosis of IM was confirmed.

### 2.4 Pathological diagnosis

The patient was ultimately diagnosed with reactive hyperplasia of the left inguinal lymph nodes with multifocal pyogenic granulomas and concurrent EBV infection. Considering the clinical presentation, this case is consistent with concurrent CSD and IM.

## 3 Discussion

### 3.1 Rare co-infection

Co-infection cases of CSD and IM remain rare. Zbinden et al. (5) documented a 19-year-old male adolescent in Switzerland, while Signorini et al. (4) reported a 7-year-old child in Italy, both involved co-infection with *Bartonella henselae* and EBV. Aparicio-Casares et al. (6) described a 5-year-old male child in Spain with disseminated *Bartonella henselae* infection complicated by osteomyelitis and hepatosplenic involvement, who was concurrently infected with EBV. However, a notable limitation of these reports was that IM diagnosis relied solely on laboratory-confirmed EBV positivity, lacking histopathological analysis.

### 3.2 Pathology-based first report

This case report described an immunocompetent adolescent male presenting with concurrent EBV and *Bartonella henselae* infection. Pathological examination of the left inguinal lymph node revealed characteristic pyogenic granulomas with Warthin-Starry silver stain-positive bacilli, suggestive of a diagnosis of CSD (7). Concurrently, the presence of EBER-positive cells by *in situ* hybridization and polyclonal B-cell lineage maturation spectrum hyperplasia (immunoblasts and plasmablasts) within the lymph node supported EBV-associated IM. Therefore, this study presented the first reported case integrating pathomorphological features with special staining techniques to confirm concurrent CSD and IM. This finding aligned with the literature-proposed hypothesis that EBV infection may promote *Bartonella henselae* dissemination through transient immunosuppression, whereby acute EBV infection activated CD4+/CD8+ T cells and natural killer (NK) cells to control EBV replication and thereby temporarily impaired immune responses against other pathogens (e.g., *Bartonella*) (8).

### 3.3 Clinical manifestations

Disseminated CSD is a systemic multi-organ disease characterized by osteomyelitis, chorioretinitis, and pneumonitis (7, 9). Studies highlighted that although hepatosplenic infectious granulomas were rare in pediatric CSD (0.3%–0.7%), their detection through abdominal ultrasound screening provided crucial evidence for differential diagnosis of FUO (10). The presence of multiple enlarged inguinal lymph nodes and FUO in this case aligned with typical descriptions of CSD in literature. Pathological examination revealed pyogenic granuloma formation within the enlarged lymph nodes. Notably, both non-contrast and contrast-enhanced CT scans of the liver and spleen showed no significant abnormalities, and systemic infection symptoms were absent, indicating that this case did not meet the criteria for disseminated infection.

### 3.4 Diagnosis based on morphology

Generally, infectious diseases are diagnosed through non-invasive testing, but this case represents the first reported co-infection of CSD and IM confirmed by pathomorphological analysis. After admission, given the prominent superficial mass but atypical accompanying symptoms, the patient underwent lymph node biopsy after excluding respiratory pathogen infections, fungal infections, and preliminary tumor marker screening. Pathological integration of dual-pathogen evidence—microscopically observed granulomas and EBER-positive *in situ* hybridization—thereby provided histological confirmation of co-infection through a morphology-based diagnosis incorporating special staining and clinical history.

### 3.5 Differential diagnoses

Notably, several differential diagnoses should be taken into consideration. Histopathological examination revealed mild follicular hyperplasia in the lymph node, increased perifollicular monocytoid cells, and numerous pyogenic granulomas within the lymphoid tissue. Upon observing these findings, primary considerations included various infectious etiologies such as CSD, lymphogranuloma venereum, tularemia, and malignant conditions like classic Hodgkin lymphoma. Immunohistochemistry showed that CD30 was focally positive, but no Hodgkin cells were observed under the microscope. Therefore, malignant classic Hodgkin lymphoma can be ruled out first. However, given the patient's 13-year-old age, the medial thigh location of enlarged lymph nodes, and accompanying febrile symptoms, CSD was prioritized. Subsequent special staining confirmed this suspicion. Importantly, morphological features inconsistent with the primary diagnosis should not be overlooked. Meticulous observation of parafollicular hyperplastic monocytoid B cells, followed by *in situ* hybridization for EBER, readily supported the diagnosis of concurrent IM.

### 3.6 Benign self-limited course

The patient had a good outcome. Despite acute-phase CSD and IM both presenting as severe systemic illnesses with prominent constitutional symptoms, their self-limiting nature ensured favorable a outcome. During hospitalization, the patient received symptomatic management—including anti-inflammatory therapy, antipyretic treatment—achieving full recovery after two weeks and being discharged in good condition.

## 4 Conclusion

In conclusion, we report an adolescent case of concurrent EBV and *Bartonella henselae* infection, emphasizing the importance of integrating serological, imaging, and pathological evaluations for both pathogens in adolescents presenting with FUO and lymphadenopathy. Notably, when encountering atypical morphological characteristics during pathological diagnosis, clinicians must correlate findings with clinical manifestations and exposure history to avoid missed diagnosis.

## Data Availability

The original contributions presented in the study are included in the article/Supplementary Material, further inquiries can be directed to the corresponding author.
